# Increased ASF1B Expression Correlates With Poor Prognosis in Patients With Gliomas

**DOI:** 10.3389/fonc.2022.912101

**Published:** 2022-07-06

**Authors:** Huaxin Zhu, Hengyang Ouyang, Xinyi Pan, Zhixiong Zhang, Jiacong Tan, Nianzu Yu, Meihua Li, Yeyu Zhao

**Affiliations:** ^1^ Department of Neurosurgery, First Affiliated Hospital of Nanchang University, Nanchang, China; ^2^ Huankui Academy, Nanchang University, Nangchang, China

**Keywords:** gliomas, ASF1B, prognosis, tcga, bioinformatics analysis

## Abstract

**Background:**

Several studies have suggested that anti-silencing function 1 B (ASF1B) can serve as a good potential marker for predicting tumor prognosis. But the values of ASF1B in gliomas have not been elucidated and further confirmation is needed.

**Methods:**

Transcriptomic and clinical data were downloaded from The Cancer Genome Atlas database (TCGA), genotypic tissue expression (GTEx), and the Chinese Gliomas Genome Atlas database (CGGA). Univariate and multivariate Cox regression analyses were used to investigate the link between clinical variables and ASF1B. Survival analysis was used to assess the association between ASF1B expression and overall survival (OS). The relationship between ASF1B expression and OS was studied using survival analysis. To investigate the probable function and immunological infiltration, researchers used gene ontology (GO) analysis, gene set enrichment analysis (GSEA), and single-sample GSEA (ssGSEA).

**Results:**

In glioma tissues, ASF1B expression was considerably higher than in normal tissues. The survival analysis found that increased ASF1B expression was linked with a poor prognosis in glioma patients. ASF1B demonstrated a high diagnostic value in glioma patients, according to a Receiver Operating Characteristic (ROC) analysis. ASF1B was found to be an independent predictive factor for OS in a Cox regression study (HR = 1.573, 95% CI: 1.053–2.350, p = 0.027). GO, KEGG, and GSEA functional enrichment analysis revealed that ASF1B was associated with nuclear division, cell cycle, m-phase, and cell cycle checkpoints. Immuno-infiltration analysis revealed that ASF1B was positively related to Th2 cells, macrophages, and aDC and was negatively related to pDC, TFH, and NK CD56 bright cells.

**Conclusion:**

A high level of ASF1B mRNA expression was correlated with a poor prognosis in glioma patients in this study, implying that it could be a reliable prognostic biomarker for glioma patients.

## Introduction

Gliomas are the most prevalent primary malignancy of the central nervous system (CNS), accounting for more than 30% of all primary brain tumors and over 80% of all malignancies. They are also the major cause of death in patients with primary CNS tumors (1, 2). The classification of gliomas mainly includes astrocytoma, anaplastic astrocytoma, oligodendroglioma, anaplastic oligodendroglioma, and glioblastoma (GBM) (3, 4). Gliomas are classified as World Health Organization (WHO) grades I–IV based on histological features: grades I and II are usually classified as low-grade gliomas (LGG), while grades III and IV are classified as high-grade gliomas (HGG) (3). Glioma subtypes have different survival rates, with LGG having a 5-year survival rate of up to 80% and HGG having a 5-year survival rate of less than 5% (5). GBM is a form of cancer that accounts for 60% of all gliomas and has a very low survival rate (3). Over the last decade, isocitrate dehydrogenase (IDH) mutation status, O6-methylguanine-DNA methyl-transferase (MGMT) promoter methylation, epidermal growth factor receptor (EGFR) alterations, and 1p19q codeletion have been identified as biomarkers and play a central role in the classification of gliomas and treatment decisions (6–9). Despite great advances in treatment, including surgery, radiotherapy, chemotherapy, and targeted therapy, nearly all malignant gliomas still experience recurrence, leading to poor prognosis (10, 11). Therefore, it is important to find novel biomarkers to provide valid and reliable survival predictions and more aggressive treatment for patients with gliomas.

Through processes such as histone-modifying enzymes, histone chaperones, and chromatin remodeling proteins, chromatin can be abnormally activated or expressed, regulating proteins that are directly associated with the genesis and spread of cancer (12, 13). Anti-silencing function 1 B (ASF1B) belongs to the histone chaperone H3/H4 family and is mainly involved in the regulation of cell proliferation (14). ASF1B expression varies with tissue, with high levels in the thymus and testis and low levels in the brain, colon, and small intestine (15). The nucleus is where the ASF1B is mostly found. In the nucleus, the cellular transcriptional co-activator HCF-1 can interact with ASF1B to regulate DNA replication, and ASF1B promotes cell proliferation by stabilizing CDK9 (16, 17). Overexpression of ASF1B has been linked to the development of tumors in various malignancies, namely, thyroid carcinoma, clear cell renal cell carcinoma, breast cancer, and lung carcinoma (18–21). Therefore, we speculate that ASF1B may also participate in the functional regulation of gliomas. However, the potential function of ASF1B in gliomas remains ambiguous.

**Figure 1:**
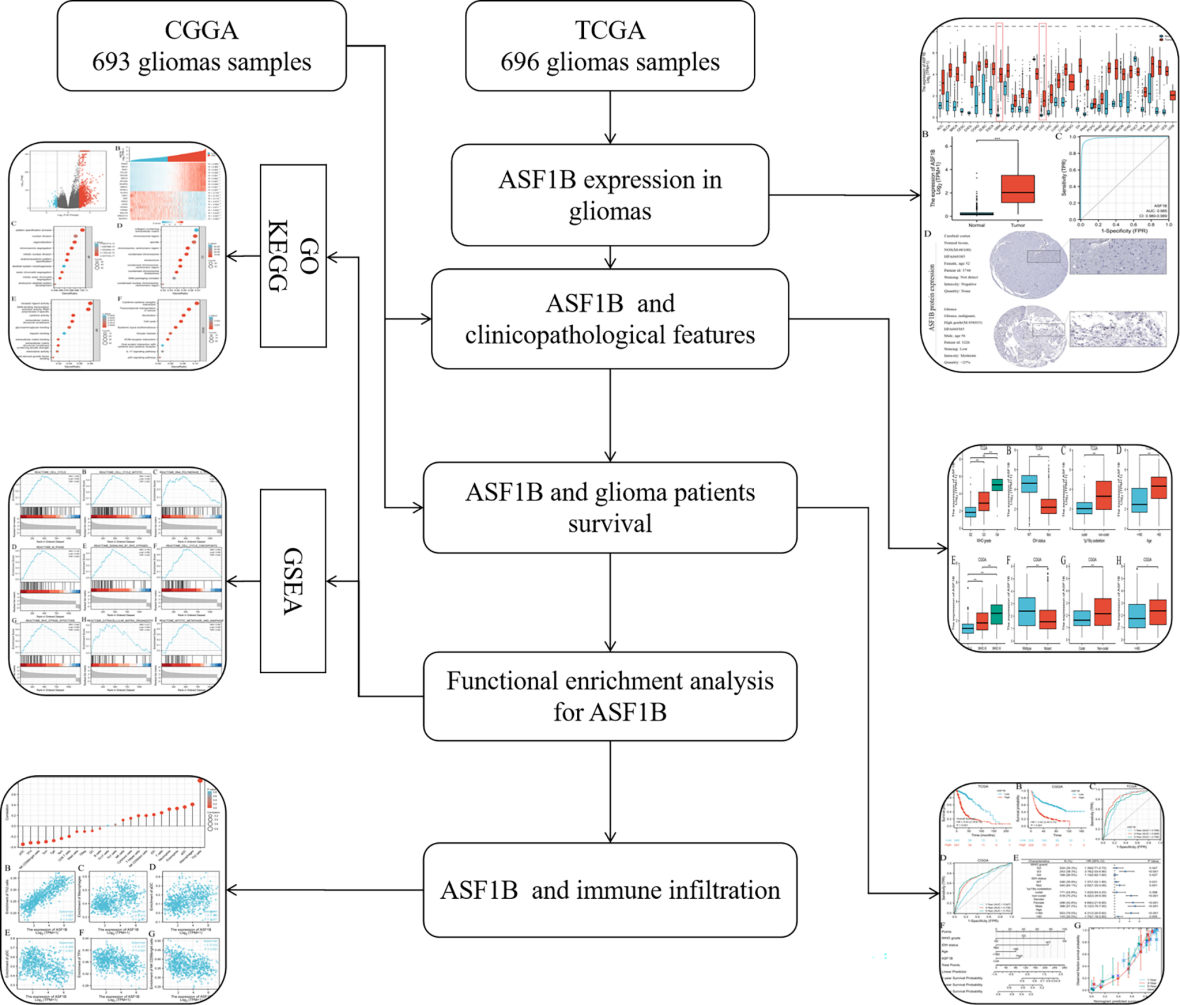
Flowchart of analysis of ASF1B in gliomas.

In this study, we first used the Cancer Genome Atlas (TCGA) database to confirm the prognostic value of ASF1B expression in glioma patients, and then validated the value using the Chinese Glioma Genome Atlas (CGGA) database. We used enrichment analysis and GSEA analysis to learn more about the biological functions and pathways that ASF1B may participate in the development of gliomas. Furthermore, we studied immune infiltration to see whether there was a link between ASF1B and immune cell infiltration and to understand the potential role of ASF1B from multiple aspects (**Figure 1**). This study confirmed the importance of ASF1B in gliomas from multilevel analysis and demonstrated that ASF1B might be a novel prognosis biomarker and therapeutic target for gliomas.

## Method

### Data Acquisition

UCSCXENA (https://xenabrowser.net/datapages/) was used to download the TCGA glioma data and the equivalent normal tissue data of GTEx by toil processing evenly. The protein expression of ASF1B in normal and glioma tissues was investigated using the Human Protein Atlas (https://www.proteinatlas.org/) database. Glioma patients were separated into two groups based on the median expression value of ASF1B: low expression and high expression. As an external validation, we downloaded glioma data from the CGGA (http://www.cgga.org.cn/) (Dataset ID: mRNAseq 693).

### A Comparison of Differentially Expressed Genes (DEGs) in Patients With Gliomas With High and Low ASF1B Expression

In this study, R package DESeq2 (1.26.0) was used to analyze low and high ASF1B mRNA expression to obtain DEGs (22). Log_2_(FC) >2.0 or log_2_(FC) <−2.0 and adj-*p*-value <0.05 was considered as the threshold for DEGs. The results of the DEGs are shown in the volcano and heat map.

### Gene Ontology (GO) Enrichment Analysis and Gene Set Enrichment Analysis (GSEA)

The R package clusterProfiler package (3.14.3) is used for GO enrichment analysis. GSEA was performed using the R package clusterProfiler too, which performed 2,000 times of gene set permutations for each analysis (23). We chose c2.cp.v7.2.symbols.gmt as the reference gene collection in the MSigDB Collections. An adj-*p*-value <0.05, false discovery rate (FDR) <0.05 and normalized enrichment score (NES) >1 were considered significant enrichment.

### Association Between the Expression of ASF1B and the Level of Immune Infiltration in Gliomas

We quantified 24 types of immune cells associated with levels of glioma immune infiltration to evaluate the correlation between immune cells and ASF1B expression by using the ssGSEA (single-sample Gene Set Enrichment Analysis) method from the GSVA package (1.34.0) in R (24). The R package is based on the TCGA database. The correlation between ASF1B and different immune cells was analyzed by Spearman’s method.

### Prognostic Model Construction and External Validation

To identify relevant prognosis markers, univariate and multivariate Cox regression analyses of clinical features were used to develop the best prognostic model. A nomogram was used to determine the likelihood of overall survival (OS). The Harrell consistency index and calibration plots were created to evaluate the dependability and correctness of the prognostic model strengths. According to ASF1B, glioma patients were divided into high- and low-risk categories. The Kaplan–Meier curve was created to indicate the difference in overall survival (OS) between the two groups. The prediction accuracy of ASF1B was evaluated using receiver operating characteristic (ROC) curves.

### Statistical Analysis

The Wilcoxon signed-rank test was used to evaluate paired samples, while the Wilcoxon rank-sum test was used to assess unpaired samples. The Kruskal–Wallis test, the Wilcoxon signed-rank test, and logistic regression were used to determine the link between clinical features and ASF1B expression. The chi-square test or Fisher’s exact test was used to examine the connection between ASF1B expression and clinical features. To generate a nomogram, researchers used univariate and multivariate Cox regression analyses of clinical variables and ASF1B to uncover relevant predictive markers. Statistical significance was defined as a P-value of less than 0.05. R (version 3.6.3) was used to conduct statistical analysis.

## Result

### Expression Level of ASF1B in Glioma Patients

**Figure 2:**
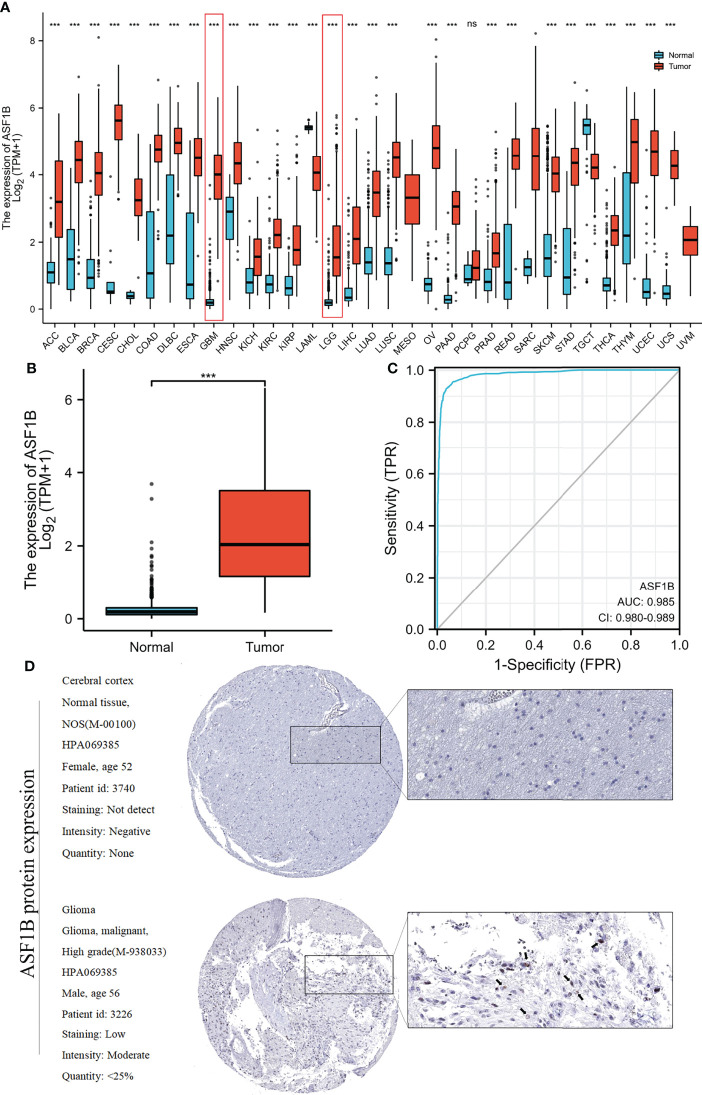
Relationship between ASF1B expression and gliomas. **(A)** ASF1B expression levels in different tumor types from the TCGA and GTEx databases. **(B)** ASF1B mRNA expression was significantly upregulated in glioma tissues compared to normal tissues. **(C)** Diagnostic value of ASF1B expression in gliomas. **(D)** ASF1B protein expression in glioma tissues determined using HPA. ***p <0.001, ns, no statistical difference.

ASF1B exhibited different expression profiles in different tumors and was significantly upregulated in glioblastoma and low-grade glioma (**Figure 2A**) (**Table 1**). We investigated ASF1B expression levels in gliomas and normal tissues and discovered that glioma tissues had considerably higher levels of ASF1B expression (p <0.001; **Figure 2B**). ROC analysis was used to determine the efficiency of ASF1B mRNA expression levels in distinguishing gliomas from normal tissues, with an estimated AUC of 0.985 (95% CI: 0.980–0.989; **Figure 2C**). Regarding the expression of ASF1B protein in gliomas, we found that the immunohistochemical analysis of ASF1B was positive in gliomas but negative in normal tissues (**Figure 2D**).

**Table 1:** Abbreviations and full names of pan-cancer.

Abbreviation	Full name	Abbreviation	Full name
ACC	Adrenocortical carcinoma	LUSC	Lung squamous cell carcinoma
BLCA	Bladder urothelial carcinoma	MESO	Mesothelioma
BRCA	Breast invasive carcinoma	OV	Ovarian serous cystadenocarcinoma
CESC	Cervical squamous cell carcinoma and endocervical adenocarcinoma	PAAD	Pancreatic adenocarcinoma
CHOL	Cholangiocarcinoma	PCPG	Pheochromocytoma and paraganglioma
COAD	Colon adenocarcinoma	PRAD	Prostate adenocarcinoma
DLBC	Lymphoid neoplasm diffuse large b-cell lymphoma	READ	Rectum adenocarcinoma
ESCA	Esophageal carcinoma	SARC	Sarcoma
GBM	Glioblastoma multiforme	SKCM	Skin cutaneous melanoma
HNSC	Head and neck squamous cell carcinoma	STAD	Stomach adenocarcinoma
KICH	Kidney chromophobe	TGCT	Testicular germ cell tumors
KIRC	Kidney renal clear cell carcinoma	THCA	Thyroid carcinoma
KIRP	Kidney renal papillary cell carcinoma	THYM	Thymoma
LAML	Acute myeloid leukemia	UCEC	Uterine corpus endometrial carcinoma
LGG	Brain lower grade glioma	UCS	Uterine carcinosarcoma
LIHC	Liver hepatocellular carcinoma	UVM	Uveal melanoma
LUAD	Lung adenocarcinoma		

### Relationship Between ASF1B Expression and Clinical Characteristics

**Figure 3:**
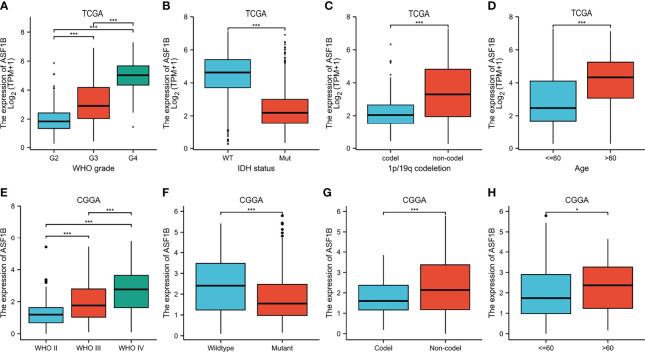
Relationship between ASF1B expression and clinicopathological features. **(A–D)** The association of ASF1B expression with WHO grade **(A)**, IDH status **(B)**, 1p/19q codeletion **(C)**, and age **(D)** in gliomas from the TCGA database. **(E, F)** The association of ASF1B expression with WHO grade **(E)**, IDH status **(F)**, 1p/19q codeletion **(G)**, and age **(H)** in gliomas from the CGGA database. *p <0.05, **p <0.01, ***p <0.001.

Clinical data on 696 and 693 glioma patients were gathered from the TCGA and CGGA databases, respectively, for this investigation (**Table 2**). According to the median mRNA expression levels of ASF1B, glioma patients were split into low and high expression groups. The relationship between ASF1B expression and several clinical features of glioma patients was investigated. It was found that there was a correlation between high ASF1B mRNA expression and higher WHO grade (p <0.001) (**Figures 3A, E**), IDH wild type (p <0.001) (**Figures 3B, F**), non-codel of 1p19q (p <0.001) (**Figures 3C, G**), and advanced age (p <0.05) (**Figures 3D, H**). Furthermore, the logistic regression analysis of ASF1B revealed a strong association between ASF1B and clinical characteristics such as WHO grade (OR = 12.664 (8.451–19.419), p <0.001), 1p/19q codeletion (OR = 4.770 (3.233–7.174), p <0.001), IDH status (OR = 14.220 (9.526–21.754), p <0.001), and age (OR = 4.412 (2.918–6.827), p <0.001) (**Table 3**).

**Table 2:** Association of ASF1B expression and clinicopathological parameters in patients with gliomas.

Characteristic	ASF1B expression in the TCGA database			ASF1B expression in the CGGA database		
	Low	High	P-value	Low	High	P-value
	n = 348	n = 348		n = 346	n = 347	
WHO grade, n (%)			<0.001			<0.001
G2	188 (29.6%)	36 (5.7%)		149 (21.5%)	39 (5.6%)	
G3	117 (18.4%)	126 (19.8%)		129 (18.6%)	126 (18.2%)	
G4	4 (0.6%)	164 (25.8%)		68 (9.8%)	181 (26.2%)	
IDH status, n (%)			<0.001			<0.001
WT	35 (5.1%)	211 (30.8%)		206 (32.1%)	150 (23.4%)	
Mut	310 (45.2%)	130 (19%)		102 (15.9%)	184 (28.7%)	
1p/19q codeletion, n (%)			<0.001			<0.001
codel	131 (19%)	40 (5.8%)		86 (13.8%)	59 (9.5%)	
non-codel	216 (31.3%)	302 (43.8%)		196 (31.5%)	282 (45.3%)	
Gender, n (%)			0.399			0.374
Female	155 (22.3%)	143 (20.5%)		141 (20.3%)	154 (22.2%)	
Male	193 (27.7%)	205 (29.5%)		205 (29.6%)	193 (27.8%)	
Age, n (%)			<0.001			0.012
≤60	315 (45.3%)	238 (34.2%)		321 (46.4%)	300 (43.4%)	
>60	33 (4.7%)	110 (15.8%)		25 (3.6%)	46 (6.6%)	
Age, median (IQR)	39 (32, 49)	54 (40, 63)	<0.001	42 (34, 50)	44 (34, 54)	0.056

**Table 3:** Logistic regression analysis of ASF1B expression.

Characteristics	Total (N)	Odds Ratio (OR)()	P-value
WHO grade (G3&G4 vs. G2)	635	12.664 (8.451–19.419)	<0.001
1p/19q codeletion (non-codel vs. codel)	689	4.770 (3.233–7.174)	<0.001
IDH status (WT vs. Mut)	686	14.220 (9.526–21.754)	<0.001
Age (>60 vs. ≤60)	696	4.412 (2.918–6.827)	<0.001
Gender (Male vs. Female)	696	1.179 (0.873–1.593)	0.284

### Prognostic Value of ASF1B Expression in Glioma Patients

**Figure 4:**
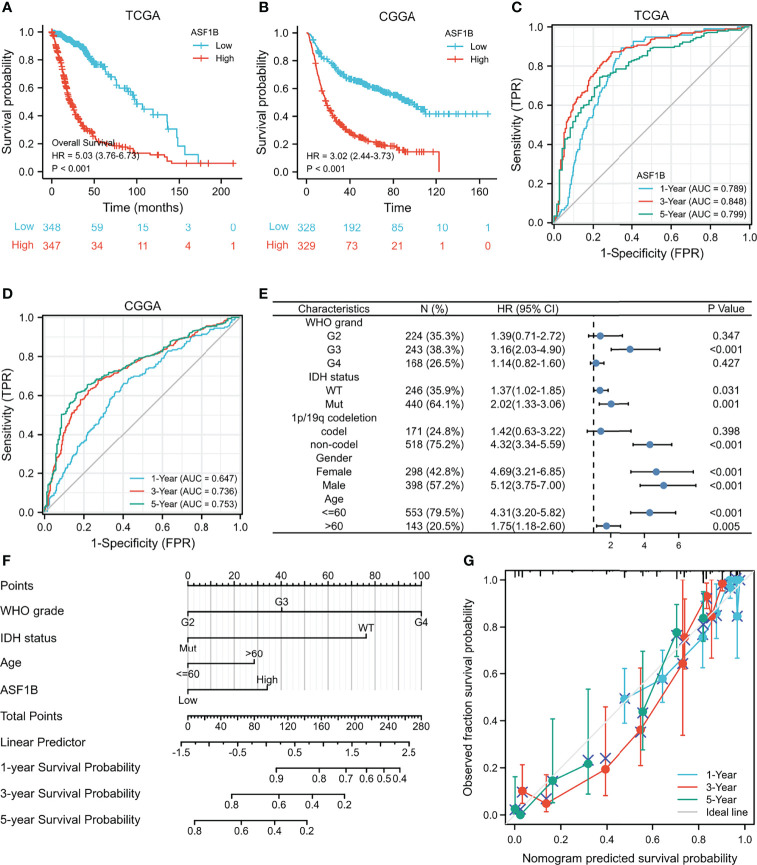
High expression of ASF1B indicates poor survival in patients with gliomas. **(A)** Kaplan–Meier survival curve analysis of overall survival (OS) showed that high ASF1B expression correlated to poor prognosis of gliomas patients from the TCGA database. **(B)** Kaplan–Meier survival curve analysis of OS showed that high ASF1B expression correlated to poor prognosis of glioma patients from the CGGA database. **(C)** Time-dependent ROC analysis of ASF1B expression in glioma patients from the TCGA database. **(D)** Time-dependent ROC analysis of ASF1B expression in glioma patients from the CGGA database. **(E)** Prognosis of ASF1B expression in subgroups of clinical features from TCGA the database. **(F)** A nomogram that integrates ASF1B and other prognostic factors in gliomas from the TCGA database. **(G)** The calibration curve of the nomogram.

Compared with the ASF1B low expression group, the ASF1B high expression group had worse OS ((HR = 5.03 (3.76–6.73), log-rank P <0.001) from the TCGA database (**Figure 4A**), (HR = 3.02 (2.44–3.73), log-rank P <0.001) from the CGGA database (**Figure 4B**) by survival analysis. ASF1B mRNA expression provides a high predictive value for the prognosis of glioma patients at 1, 3, and 5 years, according to a time-dependent ROC analysis of ASF1B expression in glioma patients (**Figures 4C, D**).

In addition, we analyzed the expression of ASF1B in different subgroups. ASF1B was highly expressed in G3 grand (HR = 3.16 (2.03–4.90), P <0.001), IDH mutant (HR = 2.02 (1.33–3.06), P = 0.001), and 1p/19q non-codeletion (HR = 4.32 (3.34–5.59), P <0.001). ASF1B expression was related to a poor prognosis (**Table 4** and **Figure 4E**).

**Table 4:** Prognostic analysis of ASF1B expression in a subset of patients with gliomas.

Characteristics	N (%)	Hazard ratio (95% CI)	P-value
WHO grand			
G2	224 (35.3%)	1.39 (0.71–2.72)	0.347
G3	243 (38.3%)	3.16 (2.03–4.90)	<0.001
G4	168 (26.5%)	1.14 (0.82–1.60)	0.427
IDH status			
WT	246 (35.9%)	1.37 (1.02–1.85)	0.031
Mut	440 (64.1%)	2.02 (1.33–3.06)	0.001
1p/19q codeletion			
codel	171 (24.8%)	1.42 (0.63–3.22)	0.398
non-codel	518 (75.2%)	4.32 (3.34–5.59)	<0.001
Gender			
Female	298 (42.8%)	4.69 (3.21–6.85)	<0.001
Male	398 (57.2%)	5.12 (3.75–7.00)	<0.001
Age			
≤60	553 (79.5%)	4.31 (3.20–5.82)	<0.001
>60	143 (20.5%)	1.75 (1.18–2.60)	0.005

We also used univariate and multivariate Cox regression to explore the clinical characteristics (**Table 5**). The high WHO grade, IDH wild type, 1p/19q non-codeletion, age >60, and high ASF1B expression were linked to poor OS according to the results of univariate Cox regression (P <0.001). Furthermore, multivariate Cox hazard regression analysis demonstrated that high WHO grade, IDH wild type, age >60, and high ASF1B mRNA expression were independent predictive variables for OS. Then, to integrate ASF1B and other prognostic factors (WHO grade, IDH status, age), we created an OS nomogram (**Figure 4F**). With a Cindex of 0.846, the calibration curve evaluated ASF1B’s nomogram performance (**Figure 4G**). All of these findings revealed that increased ASF1B expression was linked to poor prognosis in glioma patients.

**Table 5:** Univariate and multivariate analysis of clinical factors that correlate with OS of glioma patients.

Characteristics	Total (N)	Univariate analysis		Multivariate analysis	
		Hazard ratio (95% CI)	P-value	Hazard ratio (95% CI)	P-value
WHO grade	634				
G2	223	Reference			
G3	243	2.999 (2.007–4.480)	**<0.001**	1.723 (1.104–2.688)	**0.017**
G4	168	18.615 (12.460–27.812)	**<0.001**	3.880 (2.227–6.761)	**<0.001**
IDH status	685				
Mut	439	Reference			
WT	246	8.551 (6.558–11.150)	**<0.001**	3.218 (2.141–4.837)	**<0.001**
1p/19q codeletion	688				
codel	170	Reference			
non-codel	518	4.428 (2.885–6.799)	**<0.001**	1.346 (0.809–2.239)	0.252
Age	695				
≤60	552	Reference			
>60	143	4.668 (3.598–6.056)	**<0.001**	1.502 (1.103–2.046)	**0.010**
Gender	695				
Female	297	Reference			
Male	398	1.262 (0.988–1.610)	0.062	1.212 (0.923–1.593)	0.166
ASF1B	695				
Low	348	Reference			
High	347	5.029 (3.758–6.730)	**<0.001**	1.573 (1.053–2.350)	**0.027**

Values in bold indicate that the value is less than 0.05 and is statistically different.

### Functional Enrichment Analysis for High and Low ASF1B Expression in Glioma Patients

**Figure 5:**
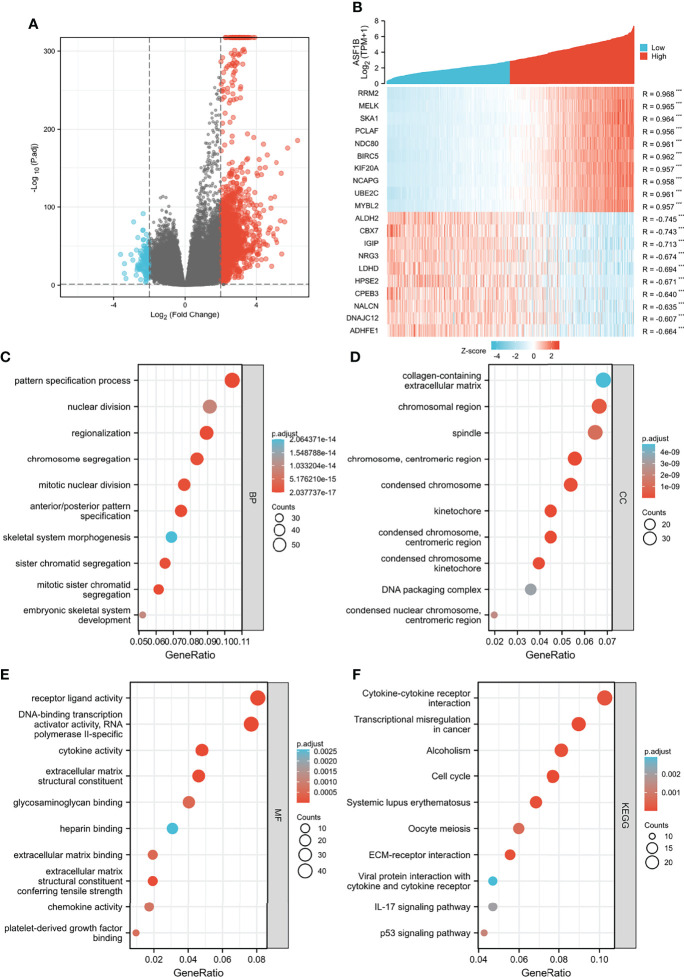
Functional enrichment analysis for ASF1B expression in glioma patients from the TCGA database. **(A)** Volcano Plot of differentially expressed genes (DEGs) screened based on ASF1B expression. **(B)** The top 10 positively correlated genes and the top 10 negatively correlated genes cotranscript with ASF1B in gliomas. **(C–F)** Enrichment analysis showed the biological processes (BP) **(C)**, cellular components (CC) **(D)**, molecular function (MF) **(E)**, and KEGG pathway analysis **(F)** of DEGs screened based on ASF1B expression. ***p <0.001.

We analyzed the DEGs between low and high expression of ASF1B groups to further explore the potential mechanisms of ASF1B that participate in glioma progression. A total of 1,660 DEGs were considered to be significantly associated with ASF1B expression, including 130 downregulated genes and 1,530 upregulated genes (log_2_FC >2 or log_2_FC <−2 and Padj <0.05) (**Figure 5A**). The top 10 positively correlated genes and the top 10 negatively correlated genes cotranscript with ASF1B are shown in the gene expression heat map (**Figure 5B**). For DEGs, we conducted the enrichment analysis of the biological process (BF), cellular component (CC), and molecular function (MF) and the Kyoto encyclopedia of genes and genomes (KEEG). Significant results of enrichment analysis are shown in **Figures 5C–F** (Further enrichment analysis and detailed information are provided in **Table S1**).

**Figure 6:**
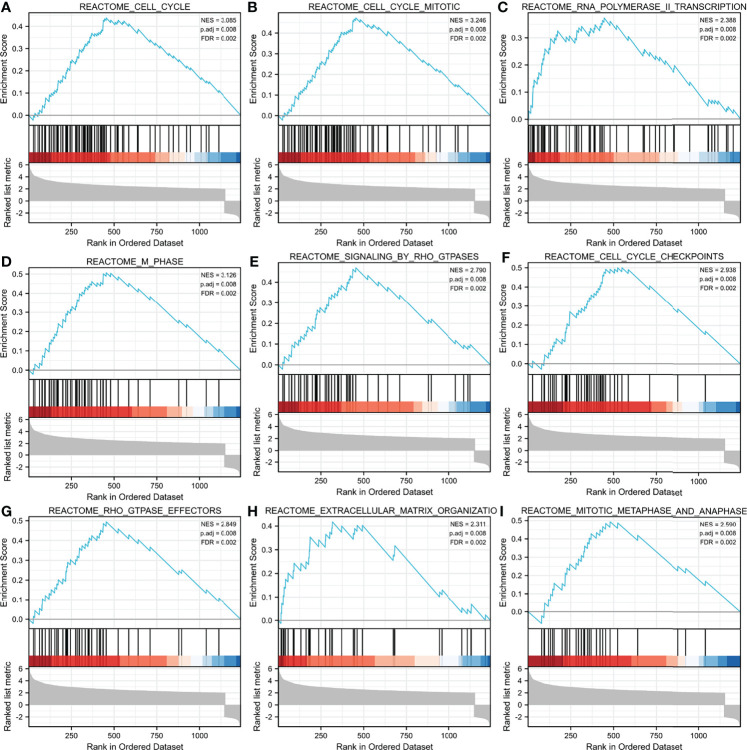
GSEA enrichment analysis results. cell cycle **(A)**,cell cycle mitotic **(B)**, RNA polymerase II transcription **(C)**, M phase **(D)**, signaling by RHO GTPases **(E)**, cell cycle checkpoints **(F)**, RHO GTPase effectors **(G)**, extracellular matrix organization **(H)**, mitotic metaphase and anaphase **(I)** were enriched mainly in ASF1B-related gliomas. NES, normalized enrichment score; FDR, false discovery rate.

Additionally, we identified key pathways associated with ASF1B by GSEA analysis and found that 106 data sets met the adjusted criteria of P-value <0.05 and FDR <0.05. (Further GSEA analysis and detailed information are provided in **Table S2**). The top 9 most significant enrichment pathways were cell cycle (**Figure 6A**), cell cycle mitotic (**Figure 6B**), RNA polymerase II transcription (**Figure 6C**), M phase (**Figure 6D**), signaling by RHO GTPases (**Figure 6E**), cell cycle checkpoints (**Figure 6F**), RHO GTPase effectors (**Figure 6G**), extracellular matrix organization (**Figure 6H**), mitotic metaphase and anaphase (**Figure 6I**) (**Table 6**).

**Table 6:** GSEA enrichment analysis results (Top 9 enrichment pathways).

Description	SetSize	EnrichmentScore	NES	p-value	p.adjust	q-values	Rank	Leading edge
CELL_CYCLE	77	0.437984556	3.08492288	0.001071811	0.007861104	0.002284467	454	tags=73%, list=36%, signal=49%
CELL_CYCLE_MITOTIC	69	0.473839241	3.245671417	0.001089325	0.007861104	0.002284467	454	tags=77%, list=36%, signal=52%
RNA_POLYMERASE_II_TRANSCRIPTION	54	0.373331996	2.387827876	0.001114827	0.007861104	0.002284467	445	tags=63%, list=36%, signal=42%
M_PHASE	48	0.506821247	3.125983655	0.001142857	0.007861104	0.002284467	454	tags=81%, list=36%, signal=54%
SIGNALING_BY_RHO_GTPASES	43	0.46987275	2.78974993	0.001164144	0.007861104	0.002284467	454	tags=77%, list=36%, signal=50%
CELL_CYCLE_CHECKPOINTS	42	0.500099904	2.937629699	0.001168224	0.007861104	0.002284467	549	tags=90%, list=44%, signal=52%
RHO_GTPASE_EFFECTORS	40	0.494357647	2.849323105	0.00118624	0.007861104	0.002284467	454	tags=80%, list=36%, signal=52%
EXTRACELLULAR_MATRIX_ORGANIZATION	35	0.417893391	2.310986332	0.001196172	0.007861104	0.002284467	317	tags=57%, list=25%, signal=44%
MITOTIC_METAPHASE_AND_ANAPHASE	30	0.493483904	2.589899346	0.001213592	0.007861104	0.002284467	480	tags=83%, list=39%, signal=52%

### Correlation Between ASF1B and Immune Cell Infiltration in Gliomas

**Figure 7:**
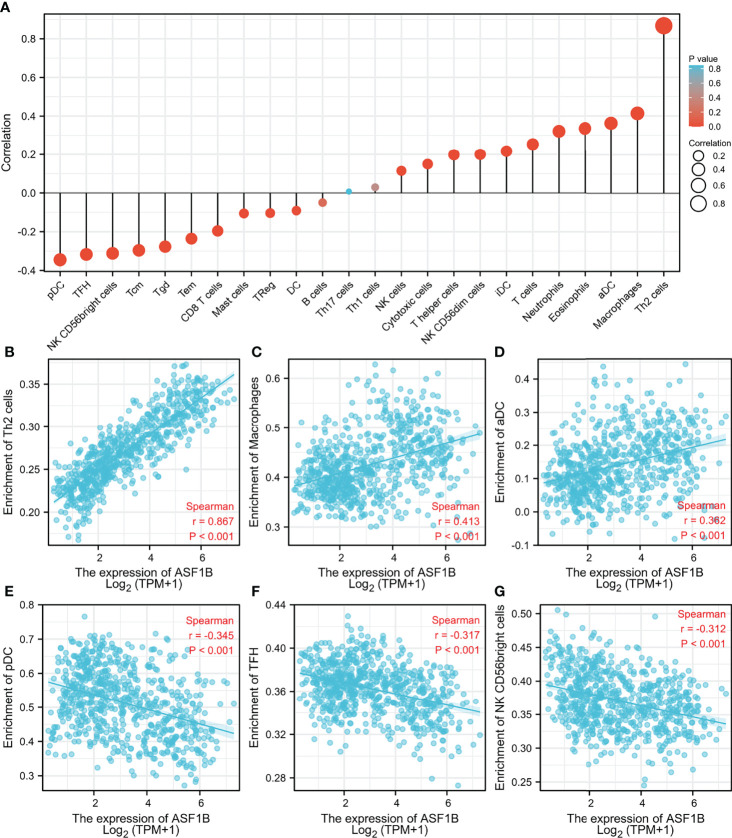
Association analysis of ASF1B expression and immune infiltration in glioma patients. **(A)** The association between ASF1B expression and 24 tumor-infiltrating lymphocytes. **(B–D)** The positive correlation of ASF1B expression with immune infiltration level of Th2 cells **(B)**, macrophages **(C)**, and aDC **(D)**. **(E–G)** The negative correlation of ASF1B expression with immune infiltration level of pDC cells **(E)**, TFH **(F)**, and NK CD56 bright cells **(G)**.

The correlation between ASF1B expression and the 24 different immune cell types was also assessed. **Figure 7A** illustrates the relationship between immune cell infiltration and ASF1B mRNA expression levels. Th2 cells (R = 0.8676, p <0.01, **Figure 7B**), macrophages (R = 0.413, p <0.01, **Figure 7C**), and aDC (R = 0.362, p <0.01, **Figure 7D**) were significantly positively correlated with ASF1B mRNA expression, whereas pDC (R = −0.345, p <0.01, **Figure 7E**), TFH (R = −0.317, p <0.01, **Figure 7F**), and NK CD56 bright cells (R = −0.312, p <0.01, **Figure 7G**) were significantly negatively correlated.

## Discussion

Gliomas are the most common kind of malignant brain tumor, and despite advances in medicine, gliomas remain incurable and have a high fatality rate (25). Patients with gliomas have a terrible prognosis, with a median survival time of 12–15 months following diagnosis (26, 27). In addition to the rapid proliferation, high invasiveness, genetic heterogeneity, and treatment refractory characteristics of glioma itself, the reasons for the low survival rate of glioma patients also include insufficient understanding of the specific molecular mechanisms that control disease progression (28, 29). As a result, new glioma biomarkers with strong prognostic value are desperately needed. The molecular biological basis of carcinoma involves aberrant mutations in genetic elements, including deletion, silencing, or overexpression of genes, changes in DNA methylation, aberrant post-translational modifications of histones and the like (30–32). ASF1 is an important histone chaperone protein that plays a role in the regulation of cellular DNA damage repair and replication and transcriptional regulation (16). ASF1 includes two isoforms, ASF1A and ASF1B, where ASF1A mainly regulates DNA repair and cellular senescence, while ASF1B mainly regulates proliferation (14, 32). Several studies indicate that ASF1B plays an important role in various cancers, namely, prostate cancer, cervical cancer, and liver cancer (17, 33, 34). However, no evidence of a link between ASF1B and glioblastoma or its prognosis has been reported.

To begin, we used transcriptome and clinical data from the TCGA and CGGA databases to investigate the predictive ability of ASF1B in gliomas, as well as probable mechanisms. ASF1B expression was shown to be considerably higher in glioma tissues, and it was linked to high-grade gliomas, advanced age, wild-type IDH, and 1p19q non-codeletion, suggesting that ASF1B plays a role in the disease process of gliomas. Meanwhile, the AUC of ASF1B in the diagnostic assessment of glioma was 0.985, indicating that ASF1B is a possible glioma biomarker. In glioma patients, upregulation of ASF1B was statistically linked to a poor prognosis. ASF1B was also found to be an independent prognostic factor in multivariate regression analysis. Considering that ASF1B is a valid and reliable independent prognostic factor, we combined ASF1B expression and clinical features to construct a nomogram to predict OS more accurately. In summary, ASF1B expression levels were positively correlated with a poorer prognosis in glioma patients.

To further identify the potential role of ASF1B in gliomas, we analyzed DEGs between low and high expression groups of ASF1B and performed functional enrichment analysis. In the GO analysis, biological processes related to cell mitosis were identified, including mitotic sister chromatid segregation, mitotic nuclear division, chromosome segregation, sister chromatid segregation, and nuclear division, etc. KEGG analysis revealed that the cell cycle and transcriptional misregulation in cancer were significantly enriched pathways. The results of GSEA revealed that ASF1B may be related to the cell cycle pathway, cell cycle mitotic pathway, RNA polymerase II transcription pathway, M phase pathway, cell cycle checkpoint pathway, mitotic metaphase, and anaphase pathway. These results were consistent with the findings of the previously published papers. According to a recent study, ASF1B can promote cervical cancer development by stabilizing CDK9, whereas inhibiting ASF1B can stop cervical cancer from growing by interrupting the cell cycle (17). ASF1B has also been shown to play a role in the formation of lung adenocarcinoma tumors, and it was discovered that ASF1B may promote tumor growth by regulating the intermediate protein BCAR1 (35). Additionally, another study pointed out that the oncogene ASF1B may be the target of inhibiting the growth of hepatocellular carcinoma cells (36). Thus, ASF1B may participate in glioma tumorigenesis by regulating cell proliferation.

According to many studies, immune cells play an important role in the microenvironment of tumors and are implicated in carcinogenesis and cancer development (37, 38). Glioma-related immune cells are an essential element of the glioma microenvironment, where they play novel roles in cancer development and anti-tumor immunity regulation (39–41). The link between ASF1B expression levels and immune cell populations was investigated to assess the amount of immune infiltration in gliomas. The findings revealed that ASF1B expression was strongly linked to immune infiltration, with the most positive correlations with Th2 cells, macrophages, and aDC and the strongest negative correlations with pDC cells, TFH, and NK CD56 bright cells. These findings suggest that ASF1B might be involved in regulating immune infiltration in the glioma tumor microenvironment.

However, there were several limitations to our study. Firstly, our study mainly relied on bioinformatics analysis and most of the data included for analysis were mined from public databases, further experimental validation is necessary to elucidate whether these predicted biological functions play a role in glioma progression and thus deepen our understanding of ASF1B in gliomas. Secondly, the number of databases used in our study was limited, and we should have further validated our findings in multiple databases. Last but not least, our study was a retrospective study and multiple limitations, including selection bias and missing data, were unavoidable. Therefore, prospective studies with large sample sizes are needed to corroborate our conclusions.

## Conclusion

In our investigation, we discovered that ASF1B was substantially expressed in glioma tissues and that it indicated a poor outcome for individuals with gliomas. The results of enrichment analyses and immune infiltration revealed that ASF1B was possibly involved in glioma tumorigenesis and development *via* modulating the cell cycle pathway, cell cycle mitotic pathway, RNA polymerase II transcription pathway, M phase pathway, cell cycle checkpoint pathway, mitotic metaphase and anaphase pathway, and immune infiltrating cells. In summary, the findings indicate that ASF1B can serve as a novel prognostic biomarker for glioma patients. Further *in vivo* and *in vitro* experiments are required to corroborate our findings

## Data Availability Statement

The original contributions presented in the study are included in the article/**Supplementary Material**. Further inquiries can be directed to the corresponding authors.

## Author Contributions

All authors listed have made a substantial, direct, and intellectual contribution to the work, and approved it for publication.

## Funding

This research was funded by the National Natural Science Foundation of China (NSFC) (No. 81860225), the Key Research and Development Plan of Jiangxi Province (No. 20203BBG73060), The Natural Science Foundation of Jiangxi Province (No. 20212BAB206029), the Science and Technology Project of Jiangxi Provincial Health Care Commission (No.20195116), and the Young Talents Research and Cultivation Foundation of the First Affiliated Hospital of Nanchang University (No. YFYPY202038).

## Conflict of Interest

The authors declare that the research was conducted in the absence of any commercial or financial relationships that could be construed as a potential conflict of interest.

## Publisher’s Note

All claims expressed in this article are solely those of the authors and do not necessarily represent those of their affiliated organizations, or those of the publisher, the editors and the reviewers. Any product that may be evaluated in this article, or claim that may be made by its manufacturer, is not guaranteed or endorsed by the publisher.
